# Epidemiology and virulence-associated genes of *Clostridioides difficile* isolates and factors associated with toxin EIA results at a university hospital in Japan

**DOI:** 10.1099/acmi.0.000086

**Published:** 2019-12-19

**Authors:** Yuta Okada, Yuka Yagihara, Yoshitaka Wakabayashi, Gene Igawa, Ryoichi Saito, Yoshimi Higurashi, Mahoko Ikeda, Keita Tatsuno, Shu Okugawa, Kyoji Moriya

**Affiliations:** ^1^​ Department of Infectious Diseases, The University of Tokyo Hospital, Bunkyo-ku, Tokyo, Japan; ^2^​ Department of Infection Control and Prevention, The University of Tokyo Hospital, Bunkyo-ku, Tokyo, Japan; ^3^​ Department of Molecular Microbiology, Graduate School of Medical and Dental Sciences, Tokyo Medical and Dental University, Bunkyo-ku, Tokyo, Japan

**Keywords:** *Clostridioides difficile*, Japan, multilocus sequence typing, epidemiology, toxin A-negative, binary toxin

## Abstract

**Introduction:**

*Clostridioides difficile* is one of the most important nosocomial pathogens; however, reports regarding its clinical and molecular characteristics from Japan are scarce.

**Aims:**

We studied the multilocus sequence typing (MLST)-based epidemiology and virulence-associated genes of isolates and the clinical backgrounds of patients from whom the isolates had been recovered.

**Methods:**

A total of 105 stool samples tested in a *C. difficile* toxin enzyme immune assay (EIA) were analysed at the University of Tokyo Hospital from March 2013 to July 2014. PCR for MLST and the virulence-associated genes *tcdA*, *tcdB*, *cdtA*, *cdtB* and *tcdC* was performed on *C. difficile* isolates meeting our inclusion criteria following retrospective review of medical records. EIA-positive and EIA-negative groups with toxigenic strains underwent clinical and molecular background comparison.

**Results:**

The toxigenic strains ST17, ST81, ST2, ST54, ST8, ST3, ST37 and ST53 and the non-toxigenic strains ST109, ST15 and ST100 were frequently recovered. The prevalence rate of *tcdA*-negative ST81 and ST37, endemic in China and Korea, was higher (11.4%) than that reported in North America and Europe, and hypervirulent ST1(RT027) and ST11(RT078) strains that occur in North America and Europe were not recovered. The linkage between the EIA results and *cdt* A/B positivity, *tcdC* deletion, or *tcdA* variation was absent among toxigenic strains. Compared with the 38 EIA-negative cases, the 36 EIA-positive cases showed that the patients in EIA-positive cases were older and more frequently had chronic kidney disease, as well as a history of beta-lactam use and proton pump inhibitor therapy.

**Conclusion:**

In Japan, the prevalence rates for *tcdA*-negative strains are high, whereas the *cdtA/B*-positive strains are rare. EIA positivity is linked to older age, chronic kidney disease and the use of beta-lactams and proton pump inhibitors.

## Introduction

*Clostridioides difficile* is an obligate anaerobic, spore-forming, Gram-positive bacterium that causes a range of gastrointestinal syndromes, from mild diarrhoea to severe pseudomembranous colitis, lethal toxic megacolon and sepsis. *C. difficile* infection (CDI) was reported for the first time in 1978 as a separate clinical entity caused by *C. difficile* [1]. Currently, CDI is widely recognized as one of the most critical healthcare-associated infections (HAI) linked to exposure to antibiotics. CDI causes significant morbidity and mortality [2, 3]. In the USA and Europe around 15 and 6 % of the HAIs are reported to be CDI, respectively [3].

The clinical symptoms of CDI are widely believed to be caused by bacterial toxins, and most pathogenic strains of *C. difficile* produce both toxin A (TcdA) and toxin B (TcdB). The genes *tcdA* and *tcdB* in the ‘PaLoc’ genomic region encode TcdA and TcdB, respectively, and some strains also produce ‘binary toxins’ (CDT) encoded by *cdtA* and *cdtB* genes in the CdtLoc genomic region [4–6]. Pathogenicity is generally defined by the presence of both *tcdA* and *tcdB* genes, but there are some exceptions to this relationship because pathogenicity has also been reported for strains with *tcdA* deletion and those producing only TcdB or CDT [7–9]. Other toxin-related genes, such as *tcdC*, *tcdE* and *tcdR* in the PaLoc region, are also thought to be involved in the regulation of toxin A and toxin B production, and the coexistence of deletions in the *tcdC* and binary toxin productivity has been reported [10–14]. Since the emergence of hypervirulent strains such as NAP1/RT027 and RT078 producing CDT, the generation of CDT has been strongly associated with increased morbidity, mortality and recurrence rates [13, 15–17]. The circulation of strains with various levels of toxigenicity has been reported worldwide, and trends differ between geographical regions [18–20].

In microbiological epidemiology studies, PCR ribotyping has historically been the more widely used method, but multilocus sequence typing (MLST) has increasingly been recognized as an equally useful molecular typing method with some advantages over PCR ribotyping in terms of ease of interpretation and the lower interlaboratory variability of test results [21]. Kuwata *et al*. performed the first microbiological epidemiology study using MLST. However, they did not examine the link between microbiological epidemiology and clinical information [22].

On the basis of this background and with the aim of adding to the few reports on the microbiological epidemiology of *C. difficile* in Japan, we investigated the toxigenicity and MLST-based epidemiology of clinical *C. difficile* isolates and analysed the clinical and microbiological backgrounds by comparing *C. difficile* toxin enzyme immunoassay (EIA)-positive and EIA-negative cases in a leading university hospital in Japan.

## Methods

### Study design

Molecular genetic analysis of *C. difficile* isolates recovered from stool samples and tested in the EIA between March 2013 and July 2014 at the University of Tokyo Hospital was conducted. In addition, we performed a retrospective analysis of clinical information in electronic medical records of the patients from whom *C. difficile* isolates were collected. *C. difficile* from a previous positive culture in the same diarrhoeal episode and patients without a confirmed record of a diarrhoeal episode or EIA result were excluded. Isolates from patients younger than 18 years old were also excluded due to the possibility of adult and paediatric CDIs having different characteristics [23]. This study was approved by the institutional Ethics Committee.

### Isolation of *C. difficile* strains

During the study period, stool samples submitted to the microbiology laboratory for EIA (C. Diff Quik Chek Complete, TechLab, Inc., Blacksburg, VA, USA) were also anaerobically cultured on cycloserine–cefoxitin–mannitol agar plates (Nissui Pharmaceutical, Co., Ltd, Tokyo, Japan) without any enrichment to enhance bacterial yield for the isolation of *C. difficile*. Anaerobic culturing was performed at 37 °C for >24 h. *C. difficile* isolates were identified by colony morphology on agar media and later confirmed by the assessment of the expression of several housekeeping genes as part of the MLST process. Colonies on agar media were routinely inoculated into skim milk and stored at −80 °C.

### Molecular assessment of *C. difficile* isolates

*C. difficile* colonies on culture agar were inoculated into sterile water and then boiled at 95 °C for 10 min to make DNA templates for subsequent PCR with an Emerald Amp PCR Master Mix kit (Takara Bio, Shiga, Japan). MLST and toxin gene analyses (*tcdA*, *tcdB*, *tcdC*, *cdtA*, *cdtB*) were performed using primers described in previous reports (Table 1) [22, 24–26]. As part of the MLST and *tcdC* analyses, the PCR amplicon underwent DNA sequencing, and the genomic sequence data of the PaLoc region of the *C. difficile* strain VPI 10463 (GenBank accession number: X92982.1) was used as reference data to detect the presence of *tcdC* deletion. Sequence types (STs) were determined based on DNA sequencing data using the PubMLST sequence query page (https://pubmlst.org/C.difficile/).

**Table 1.** List of primers used in this study

	target	Primer names	Sequence (5′-->3′)	Source
MLST	*adk*	adk1F2	CGTTGTTGGAGTTGCTTTGG	[22]
		adk1R2	TGTCAGCAACTATTTTACCTGCT	[22]
	*atpA*	atpA1F	TGATGATTTAAGTAAACAAGCTG	[25]
		atpA1R	AATCATGAGTGAAGTCTTCTCC	[25]
	*dxr*	dxr3F	GCTACTTTCCATTCTATCTG	[25]
		dxr4R	CCAACTCTTTGTGCTATAAA	[25]
	*glyA*	glyA1F	ATAGCTGATGAGGTTGGAGC	[25]
		glyA1R	TTCTAGCCTTAGATTCTTCATC	[25]
	*recA*	recA2F	CAGTAATGAAATTGGGAGAAGC	[25]
		recA2R	ATTCAGCTTGCTTAAATGGTG	[25]
	*sodA*	sodA5F	CCAGTTGTCAATGTATTCATTTC	[25]
		sodA6R	ATAACTTCATTTGCTTTTACACC	[25]
	*tpi*	tpi2F	ATGAGAAAACCTATAATTGCAG	[25]
		tpi2R	TTGAAGGTTTAACACTTCCACC	[25]
Toxin genes	*tcdA*	tcdA-F3345	GCATGATAAGGCAACTTCAGTGGTA	[24]
		tcdA-R3969	AGTTCCTCCTGCTCCATCAAATG	[24]
	*tcdB*	tcdB-F5670	CCAAARTGGAGTGTTACAAACAGGTG	[24]
		tcdB-R6079A	GCATTTCTCCATTCTCAGC	[24]
		tcdB-R6079B	GCATTTCTCCGTTTTCAGC	[24]
	*cdtA*	cdtA-F739A	GGGAAGCACTATATTAAAGCA	[24]
		cdtA-F739B	GGGAAACATTATATTAAAGCA	[24]
		cdtA-R958	CTGGGTTAGGATTATTTACTGGACCA	[24]
	*cdtB*	cdtB-F617	TTGACCCAAAGTTGATGTCTGATTG	[24]
		cdtB-R878	CGGATCTCTTGCTTCAGTCTTTATAG	[24]
	16S rDNA	PS13	GGAGGCAGCAGTGGGGAATA	[24]
		PS14	TGACGGGCGGTGTGTACAAG	[24]
	*tcdC*	tcdC-F(−17)	AAAAGGGAGATTGTATTATGTTTTC	[24]
		tcdC-R(+462)	CAATAACTTGAATAACCTTACCTTCA	[24]
	*tcdA* deletion	NK9	CCACCAGCTGCAGCCATA	[26]
		NK11	TGATGCTAATAATGAATCTAAAATGGTAAC	[26]

### Definition and analyses of clinical cases

The medical records of all the patients tested for the toxin by the EIA test were reviewed. Patients without episodes of unformed stool on electronic clinical records and those younger than 18 years old at the time of testing were excluded. Cases whose anaerobic stool cultures were negative for *C. difficile* were also excluded, and available *C. difficile* isolates were used for further molecular analysis. The clinical parameters retrieved for analysis in our study were age, sex, dates of admission and discharge if hospitalized, clinical outcome including death, length of hospitalization at EIA testing, antibiotic administration within 1 month prior to stool testing, presence of comorbidities such as diabetes mellitus, chronic kidney disease, malignancy, chemotherapy within 1 month, neutropaenia (<500 μl^−1^), daily use of immunosuppressive agents within 1 week, history of abdominal surgery, tube feeding status, and daily use of probiotics and proton pump inhibitors. White blood cell counts and serum creatinine [on the day EIA stool sample submitted if available; if not, on the closest (≤2) day after the EIA test], as well as vital signs, were also reviewed to score severity based on the definition of severity in the initial episodes of *C. difficile* infection shown in the Infectious Diseases Society of America (IDSA) clinical practice guidelines for CDI management, updated in 2017: cases with hypotension, shock, ileus, megacolon were labelled as fulminant cases, and cases with white blood cell counts ≥15,000 µl^−1^ or serum creatinine levels >1.5 mg dl^−1^ were labelled as severe cases [27]. Cases not labelled as fulminant or severe were categorized as non-severe. Clinical information for the EIA-positive and EIA-negative groups was compared, and the clinical backgrounds, outcomes and toxigenicity of the *C. difficile* isolates from the EIA-positive group were integrated with clinical information and underwent statistical analysis.

### Statistical analysis

Statistical analysis was performed using JMP software (JMP Pro 14, SAS Institute Japan, Tokyo, Japan). For categorical data and discrete measurement data analyses, the chi-squared test and Wilcoxon test were used, respectively. The Kaplan–Meier estimate was tested using the log-rank test to analyse potential differences in mortality rates between the groups. All statistical analyses were conducted with a significance level of α=0.05 (*P*<0.05).

## RESULTS

### Inclusion criteria of *C. difficile* isolates and clinical cases

A total of 105 *C*. *difficile* isolates from the same number of clinical cases were included in this study after our inclusion criteria had been applied. The 105 isolates underwent MLST and PCRs aimed at virulence-associated genes (*tcdA/tcdB*, *cdtA/B*, *tcdC* deletion). Of the 105 cases that met our inclusion criteria, 74 cases with toxigenic *C. difficile* in stool samples were divided into EIA-positive (*n*=36) and EIA-negative (*n*=38) groups, and comparative analysis between the two groups was performed.

### Microbiological epidemiology of *C. difficile* isolates

Information about ST distribution, identified clades and toxin gene presence is shown in Table 2. ST17 (*n*=12), ST81 (*n*=9), ST2ㆍST15ㆍST54ㆍST109 (*n*=7) and ST8ㆍST3ㆍST100 (*n*=4) were the most prevalent STs. Clade analysis that is commonly used to view clustering of STs with similar characteristics revealed that clade 1 strains were the most prevalent (*n*=72), followed by those of clade 4 (*n*=24). Fewer strains belonged to clades 2 and 3, and some strains could not be categorized by the data available on PubMLST. Major STs belonging to each clade are highlighted in Table 3. The proportions of isolates from EIA-positive and EIA-negative cases are also shown for commonly isolated STs in Table 4.

**Table 2.** Molecular characteristics and genetic profile of isolated *C. difficile* strains

Sequence types	Clade	No. of samples	*tcdA*	*tcdB*	*cdtA/B*	*tcdC* deletion
ST17	1	12	+	+	−	No
ST81	4	9	−	+	−	No
ST2	1	7	+	+	−	No
ST15	1	7	nt	nt	nt	nt
ST54	1	7	+	+	−	No
ST109	4	7	nt	nt	nt	nt
ST8	1	4	+	+	−	No
ST3	1	4	+(*n*=3) NT (*n*=1)	+(*n*=3) NT (*n*=1)	−	No nt
ST100	1	4	nt	nt	nt	nt
ST37	4	3	−	+	−	No
ST53	1	3	+	+	−	No
ST35	1	2	+	+	−	No
ST55	1	2	+	+	−	No
ST14	1	2	+	+	−	No
ST5	3	1	+	+	+	54 bp del
ST7	1	1	+	+	−	No
ST26	1	1	nt	nt	nt	nt
ST28	1	1	nt	nt	nt	nt
ST33	1	1	+	+	−	No
ST41	2	1	+	+	+	18 bp del
ST42	1	1	+	+	−	No
ST48	1	1	+	+	−	No
ST58	1	1	+	+	−	No
ST63	1	1	+	+	−	No
ST123	2	1	+	+	+	18 bp del
ST129	1	1	+	+	−	No
ST153	1	1	+	+	−	No
ST159	4	1	nt	nt	nt	nt
ST201	3	1	+	+	+	18 bp del
ST205	1	1	nt	nt	nt	nt
ST223	2	1	+	+	+	18 bp del
ST243	4	1	nt	nt	nt	nt
ST247	1	1	+	+	−	No
ST278	1	1	+	+	−	No
ST301	1	1	+	+	−	18 bp del
ST303	Unknown	1	nt	nt	nt	nt
ST304	Unknown	1	+	+	−	No
ST400	1	1	+	+	+	No
ST401	4	1	nt	nt	nt	nt
ST402	Unknown	1	nt	nt	nt	nt
ST403	4	1	nt	nt	nt	nt
ST404	1	1	+	+	−	No
ST405	4	1	nt	nt	nt	nt
ST406	1	1	nt	nt	nt	nt
ST407	Unknown	1	nt	nt	nt	nt
ST408	1	1	nt	nt	nt	nt

NT: negative due to lack of PaLoc

**Table 3.** Most common STs in clades 1–4

	Total	Major STs: toxigenic strains	Major STs: non-toxigenic strains
Clade 1	72	ST17 (*n*=12, 16.7%), ST2ㆍST54 (*n*=7, 9.7%)	ST15 (*n*=7, 9.7%) ST100 (*n*=4, 5.6%)
Clade 4	24	ST81 (*n*=9, 37.5%) ST37 (*n*=3, 12.5%)	ST109 (*n*=7, 29.2%)
Clade 2	3	ST41, ST123, ST223 (*n*=1, 33.3%)	–
Clade 3	2	ST5, ST201 (*n*=1, 50%)	–
unknown	4	ST304 (*n*=1, 25%)	ST303, ST402, ST407 (*n*=1, 25%)

**Table 4.** STs and clades detected in the EIA-positive and EIA-negative groups

	Total nos	EIA-positive	EIA-negative
Toxigenic			
ST17/Clade 1	12	8	4
ST81/Clade 4	9	3	6
ST2/Clade 1	7	1	6
ST54/Clade 1	7	5	2
ST8/Clade 1	4	2	2
Others	35	17	18

### Relationships between toxigenicity, *cdtA/B*, *tcdA* variation and *tcdC* deletion with ST

As shown in Table 2, out of the 105 isolates studied, 74 were toxigenic and 31 were non-toxigenic. All toxigenic strains were confirmed to possess the *tcdC* gene, whereas non-toxigenic strains did not express it. The presence of *cdtA/cdtB* genes, *tcdA* deletions and *tcdC* deletions was confirmed in 5, 12 and 6 isolates, respectively. Of the six isolates with the *tcdC* deletion, five were *cdtA/cdtB*-positive, whereas one was *tcdA*+/*tcdB*+ and *cdtA/B*-negative. Except for ST3, no discrepancy in toxin gene patterns was observed between isolates in the same ST. In ST3, three isolates were toxigenic strains, whereas one isolate was non-toxigenic.

### Toxin EIA test results and *tcdC* deletion, binary toxin and toxin A variation

A total of 74 clinical cases were confirmed to be toxigenic, and the toxin EIA test was positive in 36 cases and negative in 38 cases. The most frequently found strains in the EIA-positive and EIA-negative groups are listed in Table 4. No significant differences between the two groups regarding the prevalence rates of *tcdA*-negative variants, cdtA/B-positive strains and strains with *tcdC* deletion in the EIA-positive and EIA-negative groups were found (Table 5).

**Table 5.** EIA positivity and prevalence of *cdtA/B*, *tcdC* deletion and *tcdA* deletion in *C. difficile* isolates

	EIA-positive (*n*=36)	EIA-negative (*n*=38)	*P* value
*cdt* A/B-positive	5 (13.9 %)	1 (2.6 %)	0.0762
*tcdC* deletion (18 or 54 bp)	4 (11.1 %)	2 (5.3 %)	0.3570
*tcdA* variant	3 (8.3 %)	9 (23.7 %)	0.0734

### Comparison of clinical background between EIA-positive and EIA-negative patients

Of the 74 clinical cases finally included in our clinical analysis, 36 cases were EIA-positive and 38 cases were EIA-negative. The EIA-positive patients were slightly but significantly older than the EIA-negative patients (71.8±12.4 vs 62.4±17.6 years, *P*=0.0106) and were significantly more frequently in the hospital for 3 days or longer (97.2 % vs 78.9 %, *P*=0.0162), while a significantly higher proportion of patients in the EIA-positive group had chronic kidney disease (41.7 % vs 18.4 %, *P*=0.0288). The proportion of patients who had used antibiotics within 30 days of the EIA test (100 % vs 89.5 %, *P*=0.0453), and beta-lactams in particular (100 % vs 78.9 %, *P*=0.0097), was also higher in the EIA-positive group than in the EIA-negative group. The proportion of patients who were on a proton pump inhibitor within 1 week before the EIA stool testing date was also higher in the EIA-positive group than in the EIA-negative group (83.3 % vs 62.2 %, *P*=0.0426).

The other clinical factors analysed in our study did not differ significantly between these groups. A complete list of the clinical factors analysed in this study is provided in Table 6.

**Table 6.** Comparison of patients EIA-positive and EIA-negative for *C. difficile* toxin

	EIA-positive (*n*=36)	EIA-negative (*n*=38)	*P* value
Age (mean±1sd)	71.8±12.4	62.4±17.6	0.0106
Male sex	17 (47.2 %)	15 (39.5 %)	0.5013
Hospital stay for 3 days or longer	35 (97.2 %)	30 (78.9 %)	0.0162
Diabetes mellitus	9 (25.0 %)	6 (15.8 %)	0.3246
Chronic kidney disease	15 (41.7 %)	7 (18.4 %)	0.0288
Inflammatory bowel disease	1 (2.8 %)	4 (10.5 %)	0.1844
Patients with active malignancy	16 (44.4 %)	15 (39.5 %)	0.6649
Haematological malignancy	9 (25.0 %)	7 (18.4 %)	0.4920
Solid organ malignancy	7 (19.4 %)	8 (21.1 %)	0.8634
Chemotherapy within 1 month	8 (22.2 %)	9 (23.7 %)	0.8812
Neutropaenia (<500 µl^−1^)	1 (2.8 %)	3 (7.9 %)	0.3306
Use of immunosuppressive agents	10 (27.8 %)	14 (36.8 %)	0.4051
Corticosteroids	10 (27.8 %)	14 (36.8 %)	0.4051
Non-steroid	4 (11.1 %)	4 (10.5 %)	0.9355
Abdominal surgery within 1 month	5 (13.9 %)	3 (7.9 %)	0.4065
History of abdominal surgery	15 (41.7 %)	9 (23.7 %)	0.0986
Tube feeding within 1 week	5 (14.3 %)	4 (10.5 %)	0.6255
Antibiotic treatment			
Use of antibiotics within 4 weeks	36 (100.0 %)	34 (89.5 %)	0.0453
Beta-lactams	36 (100.0 %)	30 (78.9 %)	0.0036
Third- or fourth-generation cephalosporins	12 (33.3 %)	12 (31.6 %)	0.8720
Betalactam/beta lactamase inhibitor	15 (41.7 %)	13 (34.2 %)	0.5086
Carbapenems	6 (16.7 %)	4 (10.5 %)	0.4400
Fluoroquinolones	4 (11.1 %)	2 (5.3 %)	0.3570
Other antibiotics	16 (44.4 %)	16 (42.1 %)	0.8391
Proton pump inhibitor therapy within 1 week	30 (83.3 %)	23 (62.2 %)	0.0426
Use of probiotics within 1 week	8 (22.2 %)	10 (27.0 %)	0.6339

## Discussion

### Microbiological epidemiology and virulence-associated genes

This study is one of the most thorough microbiological analyses of the epidemiology and virulence factors of *C. difficile* isolates in Japan, and also provides an insight into the clinical background of cases from which *C. difficile* were isolated through comparisons based on toxin presence revealed by EIA.

Epidemiological analysis of the *C. difficile* isolates in our study is essential as MLST-based reports from Japan are scarce. Our study results are consistent with those from previous studies reporting that ST17 (corresponding to RT018), ST2 and ST54 are prevalent strains in Japan [22, 28–30]. The relatively high prevalence rates for *tcdA*-negative/*tcdB*-positive strains ST81 and ST37 in our study is in the range of previously reported prevalence rates of 10–30 % in Asian countries, including China and Korea [31–34], and higher than that reported in non-Asian regions, where only rare outbreaks of *tcdA*-negative/*tcdB*-positive strains have been reported [35, 36]. In the USA and Europe, the fractions of *tcdA*-negative/*tcdB*-positive strains recovered from clinical stool samples were reported to be less than 3–5 %, respectively [19, 37].

The rarity of binary toxin-producing strains, including ST1(RT027) or ST11(RT078) in our study, is consistent with reports from Japan’s neighbouring countries showing the same tendency compared with North America or Europe [22, 31–34]. The binary toxin-producing ST41, ST123 and ST223 strains in our study are also reported to cause severe CDI [25, 38]. In previous reports, it was also shown that these strains share *cdtA/B*, 18 bp deletion in *tcdC* and fluoroquinolone resistance with the ST1/RT027 strain in the same clade (2) [25, 38–40], but differences between these strains and the ST1/RT027 strain, such as the structure and cytotoxicity of TcdB and toxin productivity, have also been reported [38, 41]. An outbreak of these strains may have a significant impact in countries where ST1/RT027 strains are rarely found, and further insight into the pathogenicity of these strains is warranted.

With respect to non-toxigenic strains, the relatively high prevalence rate of non-toxigenic strains ST15, ST109 and ST100 was consistent with a previous study performed in Japan [22]. Non-toxigenic strains are of interest because there are reports concerning the protective effect of colonization by non-toxigenic strains against CDI [42], and some preclinical and clinical studies have shown that the administration of certain selected non-toxigenic *C. difficile* strains could play a defensive role against CDI development [43–46]. Our epidemiological data on non-toxigenic strains may contribute to future treatment or prevention measures against CDI.

The discrepancy in toxigenicity is known to be observed among isolates in the same strain, and the discrepancy in toxigenicity in ST3 in our study was also shown previously [33]. Other strains, including ST109 and ST100, have also been shown to encompass both toxigenic and non-toxigenic strains [47, 48], and this issue warrants further evolutionary study [49].

### Comparison of EIA-positive and EIA-negative groups

Comparison between EIA-positive and EIA-negative cases was performed as in daily practice. Our hospital and many other hospitals utilize EIA test results for CDI diagnosis and management. Previous studies have shown that EIA positivity is positively linked to the presence of *cdtA/B* [50, 51] and *tcdC* deletion [52]. However, given the limited statistical power of our study, we could only suggest that *cdt*A/B positivity correlates with EIA positivity. The proportion of *tcdA*-negative isolates was not significantly different between the two groups, which is consistent with previous findings [33, 53].

The comparison between the EIA-positive and EIA-negative groups highlighted advanced age, hospital stay of longer than 3 days, chronic kidney disease, and use of beta-lactam antibiotics and proton pump inhibitors as factors that correlated with the positive EIA result. These factors are reportedly recognized as critical elements that increase the risk of CDI development [54–58]. Although seemingly contradicting the results of two previous studies [59, 60], in our study, the history of probiotic use was not linked to a higher rate of EIA-positive results.

### Limitation

The small number of clinical cases (partly due to the exclusion of paediatric cases) and the limited availability of *C. difficile* isolates due to the nature of our single institution-based study are the limitations of our study.

### Conclusion

Our study makes a significant contribution to the field as one of the first MLST-based epidemiological studies performed in Japan. The relatively high prevalence of toxin A-negative/B-positive strains and the low prevalence of hypervirulent binary toxin-producing strains observed in this study were similar to the epidemiological data reported in previous studies in Japan. Older age, recent use of beta-lactam antibiotics and proton pump inhibitors, and more prolonged hospitalization in the EIA-positive group were demonstrated in our study, whereas no correlations between EIA positivity and microbiological virulence-associated factors were found.
